# Prognostic value of automated bone scan index for predicting overall survival among bone metastatic castration resistant prostate cancer patients treated with radium‐223

**DOI:** 10.1002/bco2.43

**Published:** 2020-09-05

**Authors:** Yasuhide Miyoshi, Sohgo Tsutsumi, Takashi Kawahara, Masato Yasui, Koichi Uemura, Shuko Yoneyama, Yumiko Yokomizo, Narihiko Hayashi, Masahiro Yao, Hiroji Uemura

**Affiliations:** ^1^ Department of Urology and Renal Transplantation Yokohama City University Medical Center Yokohama Japan; ^2^ Department of Urology Yokohama City University School of Medicine Yokohama Japan

## Abstract

**Introduction:**

The objective of this study was to evaluate automated bone scan index (aBSI) as a prognostic biomarker for overall survival (OS) in bone‐metastatic, castration‐resistant prostate cancer (mCRPC) patients treated with radium‐223 (Ra‐223).

**Materials and methods:**

We identified 42 men treated with Ra‐223 for mCRPC. We investigated aBSI as an independent prognostic factor by multivariate analysis. Moreover, we evaluated the prognostic value of the aBSI after 12 weeks after the first cycle of Ra‐223 administration and aBSI change from baseline to after 12 weeks (ΔBSI).

**Results:**

Median baseline PSA and aBSI were 42.8 ng/mL and 1.5%, respectively. Median OS was 20.7 months. Multivariate analysis showed that baseline aBSI was a significant prognostic factor for OS. The aBSI at 12 weeks after first Ra‐223 administration also exhibited significant prognostic value for OS, while we found no evidence of prognostic value for ΔBSI.

**Conclusions:**

Baseline aBSI may be a significant prognostic factor for OS in bone‐metastatic CRPC patients treated with Ra‐223.

## 1 INTRODUCTION

Prostate cancer (PC) is the second most frequently occurring cancer with 1,276,106 new cases reported worldwide in 2018.^1^ Metastatic PC is an androgen‐dependent tumor that initially responds to androgen deprivation therapy (ADT), however, most patients fail ADT and develop castration‐resistant PC (CRPC).^2^ Currently, a number of agents and therapies are used for the treatment of metastatic CRPC (mCRPC). Radium‐223 (Ra‐223), an alpha emitter that selectively targets bone metastases, is a life‐prolonging agent used to treat mCRPC.^3^ A phase III, placebo‐controlled randomized clinical trial, ALSYMPCA, demonstrated that six cycles of Ra‐223 resulted in a 30.0% risk reduction in overall survival (OS) compared with placebo among men with bone metastatic CRPC. Furthermore, Ra‐223 also significantly improved the time to first symptomatic skeletal event which strongly enhances patient quality of life.^4^

As described above, Ra‐223 exhibits a strong OS benefit for men with mCRPC, however, robust prognostic or predictive biomarkers have not been established.^5^ Automated bone scan index (aBSI) is an objective tool for quantifying bone metastasis burden in various cancers. The aBSI is a method of expressing the tumor burden in bone as a percent of the total skeletal mass, based on reference values for skeletal masses.^6^

Previous studies reported that aBSI could be used as an imaging biomarker for predicting survival in men with bone‐metastatic hormone‐naïve PC and CRPC.^6^, ^7^, ^8^, ^9^, ^10^, ^11^, ^12^, ^13^, ^14^, ^15^, ^16^, ^17^, ^18^, ^19^, ^20^, ^21^, ^22^, ^23^ However, the prognostic value of aBSI among men treated with Ra‐223 remains unclear. We evaluated aBSI as a prognostic imaging biomarker to predict OS in a cohort of bone metastatic CRPC patients treated with Ra‐223.

## 2 PATIENTS AND METHODS

### 2.1 Study design, patients, and treatment

We identified consecutive 42 men treated with Ra‐223 for bone metastatic CRPC at the Yokohama City University Medical Center between 2012 and 2017. Some patients took part in Japanese clinical trial for Ra‐223 before Japanese approval. All patients had a histologically confirmed diagnosis of prostate adenocarcinoma. In addition, all patients received ADT (luteinizing hormone‐releasing hormone [LHRH] with bicalutamide or flutamide) as an initial treatment, and no patient received upfront abiraterone, apalutamide, enzalutamide, or docetaxel for metastatic hormone‐naïve prostate cancer.

Metastatic sites were evaluated by computed tomography (CT) and bone scan using 99‐technetium methylene diphosphonate (MDP) within 1 month before Ra‐223 treatment. Patients with distant metastases, except bone, and/or regional lymph node metastases greater than 4 cm, were excluded from the study. Ra‐223 was administered at 55 kBq (1.49 microcurie)/kg intravenously every 4 weeks up to six cycles. LHRH agonist or antagonist was administrated with Ra‐223 in all patients.

Clinical data including patient age, Gleason scores, time to CRPC from initial androgen deprivation therapy for hormone‐naïve PC, previous treatment with docetaxel, baseline serum prostate‐specific antigen (PSA), baseline aBSI, Eastern Cooperative Oncology Group performance status, pain, concomitant use of bone‐modified agents (BMA), previous use and concomitant use of bicalutamide or flutamide, abiraterone acetate (ABI) or enzalutamide (ENZ), and survival were collected from electronic medical records. The extent of disease on bone scan (EOD) classifications^24^ was measured manually by a single investigator (YM).

Thirty‐one out of 42 (73.8%) patients also received a bone scan at 12 weeks after Ra‐223 treatment and we collected the aBSI data after 12 weeks of Ra‐223 induction. In addition, in 15 of the 31 patients, we collected aBSI data 24 weeks after the induction of Ra‐223.

The Gleason scores were determined according to the 2005 International Society of Urologic Pathology Gleason grading.^25^ Serum PSA levels were measured using the Elecsys PSA Assay (Roche Diagnostics, Basel, Switzerland). Pain was defined according to patients’ complaint regardless of painkiller use. In Japan, Ra‐223 have been approved for not only mCRPC patients with pain, but also without pain.

After Ra‐223 treatment failure, all patients were administered LHRH agonist or antagonist continuously and subsequently treated according to their respective physician's treatment strategy. In the terminal stage, palliative therapy and pain control with morphine, and palliative external‐beam radiation were used as appropriate.

### 2.2 aBSI

A total of 42 patients received a bone scan within 1 month before Ra‐223 treatment and 31 out of 42 (73.8%) patients also received a bone scan at 12 weeks after the first cycle of Ra‐223 administration.

Baseline aBSI levels, aBSI levels at 12 weeks after the first cycle of Ra‐223 administration, and aBSI change from baseline to 12 weeks after the first cycle of Ra‐223 administration (ΔBSI) was obtained. Moreover, in 15 patients, we compared aBSI at 12 weeks and 24 weeks after Ra‐223 induction.

The aBSI was calculated based on a bone scan using 99‐technetium MDP (FUJIFILM Toyama Chemical Co., Ltd, Tokyo, Japan) by BONENAVI version 2.^26^ The automated method for the analysis of anterior and posterior whole‐body bone scan images was done as previously described.^9^

### 2.3 Statistical analysis

Continuous variables were summarized as median and range. The correlation between aBSI and EOD classifications was analyzed using Spearman's correlation coefficient.

For evaluating the prognostic value of baseline aBSI, we retrospectively investigated the correlation between baseline clinical factors including patient age, Gleason scores, time to CRPC, previous treatment with docetaxel, baseline PSA, baseline aBSI, and OS after Ra‐223 treatment by univariate and multivariate analysis using the Cox proportional hazard model with stepwise regression analysis.

For evaluating the prognostic value of both aBSI at 12 weeks after first cycle of Ra‐223 administration and ΔBSI, univariate and multivariate analysis by the Cox proportional hazard model was also used to analyze the correlation between aBSI at 12 weeks after treatment, ΔBSI, and OS.

The relative risks and 95% confidence intervals (CIs) were calculated. Running variables were divided into binary variables according to the median. ΔBSI was divided into two categories as an increase and decrease from baseline.

The Kaplan‐Meier product limit was used to estimate survival distribution. The log‐rank test was used for the analysis of survival differences. All statistical tests were two‐sided, and the significance level was defined as *α* = .05. All analyses were conducted using IBM SPSS Statistical Software for Windows, version 24 (IBM Co, Ltd, Armonk, NY, USA) and EZR version 3.5.2. (Saitama Medical Center, Jichi Medical University, Saitama, Japan).^27^

## 3 RESULTS

### 3.1 Patient characteristics

Patient characteristics are listed in Table 1. Thirty out of 42 (71.4%) patients had a metastatic lesion identified at the initial diagnosis, before developing CRPC, and 12 out of 42 (28.6%) patients received local therapy including prostatectomy and external beam radiation therapy as an initial treatment, before developing CRPC.

**TABLE 1:** Patient characteristics

Variables		
Gleason scores at initial biopsy	≤7	8 (19.0%)
	8‐10	33 (78.6%)
	Unknown	1 (2.4%)
Median age (range), years		75.5 (57.4‐87.0)
Median time to CRPC (range), months		11.2 (1.8‐119.9)
Median aBSI (range), %		1.5 (0.0‐11.5)
Median PSA (range), ng/mL		42.8 (0.3‐1795.0)
Mets of extra‐bone, n (%)	Yes	9 (21.4%)
Previous use of docetaxel, n (%)	Yes	9 (21.4%)
ECOG performance status, n (%)	0‐1	40 (95.2%)
	2	1 (2.4%)
	Unknown	1 (2.4%)
Pain, n (%)	Yes	27 (64.3%)
Concomitant use of BMA, n (%)	Yes	25 (59.5%)
Previous use of BIC/FLU*, n (%)	Yes	42 (100%)
Concomitant use of BIC/FLU, n (%)	Yes	0 (0.0%)
Previous use of ABI/ENZ**, n (%)	Yes	13 (41.0%)
Concomitant use of ABI/ENZ, n (%)	Yes	9 (21.4%)

Abbreviations: ABI, abiraterone acetate; aBSI, automated bone scan index; BIC, bicalutamide; BMA, bone‐modified agent; CRPC, castration‐resistant prostate cancer; ECOG, Eastern Cooperative Oncology Group; ENZ, enzalutamide; FLU, flutamide; Mets, metastases; PSA, prostate‐specific antigen.

* Previous use of BIC/FLU indicate administration of BIC/FLU for hormone‐naïve prostate cancer;

** Previous use of ABI/ENZ indicate administration of ABI/ENZ for CRPC.

**FIGURE 1:**
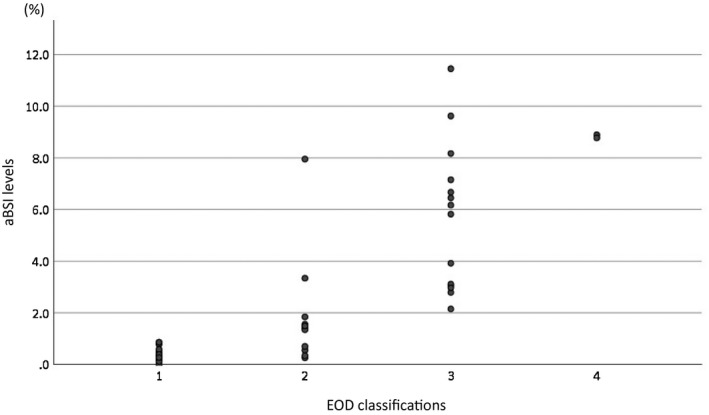
The correlation between aBSI and EOD classifications which counted manually. There was a significant correlation between those two variables. The correlation coefficient was 0.85

The correlation between aBSI and EOD classifications is shown in Figure 1; there was a significant correlation between these two variables (rs = 0.85).

### 3.2 aBSI and survival

The median observation period was 11.7 months. Of the total 42 patients, 25 (59.5%) completed 6 cycles of Ra‐223 treatment. The reasons for the remainder of the patients receiving five or less administrations of Ra‐223 were: cancer progression in 11 patients, adverse event in 2 patients, patient preference in 2 patients, and the reason was unknown in 2 patients. Three patients died within 6 months after Ra‐223 induction.

**FIGURE 2:**
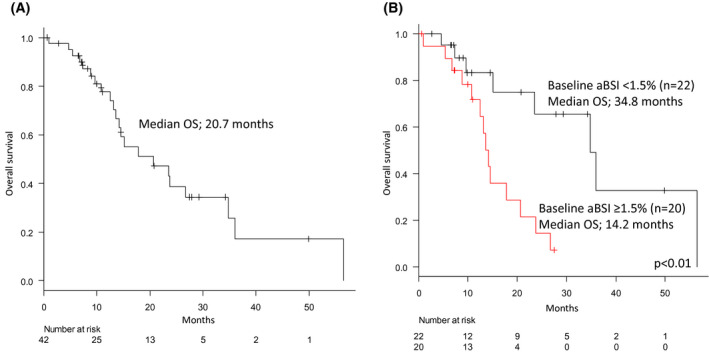
A, Kaplan‐Meier curve for overall survival (OS) among men treated with Radium‐223 (n = 42). Median OS was 20.7 months. B, Kaplan‐Meier curve for overall survival (OS) among men treated with Radium‐223 according to baseline aBSI levels. Median OS among men with < 1.5% baseline aBSI was 34.8 months (n = 22) and 14.2 months in men with ≥ 1.5% baseline aBSI (n = 20). There was a significant OS difference between men with < 1.5% baseline aBSI and with ≥ 1.5% baseline aBSI (*P* < .01)

Twenty‐two patients (52.4%) died including 20 (47.6%) cancer deaths occurring at the time of analysis. The median OS in the entire cohort was 20.7 months (Figure 2A).

Univariate analysis showed that baseline PSA (>42.8 ng/mL vs ≤ 42.8, HR 2.82, 95.0%CI 1.12‐7.12, *P* = .03), and baseline aBSI (>1.5% vs ≤ 1.5, HR 4.31, 95.0%CI 1.53‐12.15, *P* = .01) were significant prognostic factors for OS (Table 2). Multivariate analysis showed that only baseline aBSI (>1.5% vs ≤ 1.5, HR 3.72, 95.0%CI 1.17‐11.79, *P* = .03) was a significant prognostic factor for OS (Table 2). Figure 2B showed Kaplan‐Meier curve for OS among men treated with Ra‐223 according to baseline aBSI levels. Median OS among men with < 1.5% baseline aBSI was 34.8 months (n = 22) and 14.2 months in men with ≥ 1.5% baseline aBSI (n = 20). There was a significant OS difference between men with < 1.5% baseline aBSI and with ≥ 1.5% baseline aBSI (*P* < .01).

**TABLE 2:** Evaluation of baseline aBSI for predicting OS in patients treated with radium‐223 by univariate and multivariate analysis

	Univariate				Multivariate			
	*P*‐value	HR	95.0% CI		*P*‐value	HR	95.0% CI	
			Lower	Upper			Lower	Upper
Age > 75.5 years vs ≤ 75.5	.10	0.46	0.19	1.15	.28	0.54	0.18	1.66
Time to CRPC < 11.2 months vs ≥ 11.2	.91	1.05	0.44	2.52	.86	1.11	0.36	3.42
Previous use of docetaxel, Yes vs no	.87	0.91	0.32	2.59	.71	1.27	0.35	4.62
Baseline PSA > 42.8 ng/mL vs ≤ 42.8	.03	2.82	1.12	7.12	.23	2.06	0.64	6.68
Gleason sum 9‐10 vs 6‐8	.73	1.24	0.35	4.40	.85	1.15	0.27	4.92
Baseline aBSI > 1.5% vs ≤ 1.5	.01	4.31	1.53	12.15	.03	3.72	1.17	11.79

Abbreviations: aBSI, automated bone scan index; ALP, alkaline phosphatase; CI, confidence interval; CRPC, castration‐resistant prostate cancer; HR, hazard ratio; PSA, prostate‐specific antigen.

Furthermore, we evaluated the prognostic value of both aBSI at 12 weeks after the first cycle of Ra‐223 administration and ΔBSI. At the 12‐week mark, 15 patients had received two cycles of Ra‐223 (before 3 cycles of Ra‐223), and the remaining sixteen patients received three cycle of Ra‐223. The aBSI was obtained from 10 to 15 weeks after Ra‐223 induction at the 12‐week mark.

**FIGURE 3:**
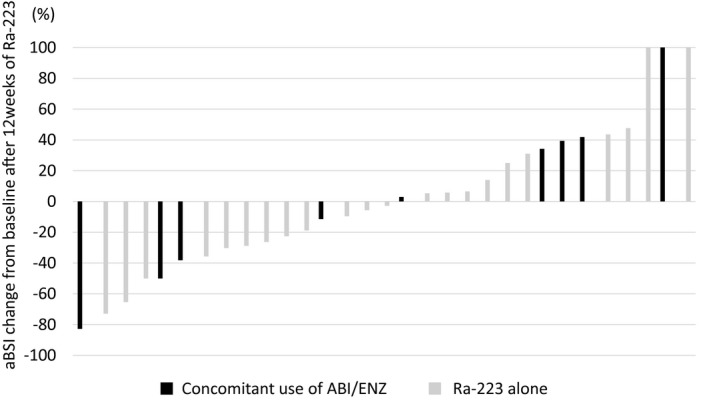
Waterfall plot of bone scan index change from baseline after 12 weeks of Radium‐223 (Ra‐223) treatment (n = 31). Black and gray bars indicate the patients treated with Ra‐223 combined with abiraterone acetate (ABI) or enzalutamide (ENZ) and without use, respectively

The median aBSI at 12 weeks after treatment was 2.0%. Figure 3 shows the waterfall plot for ΔBSI. The median ΔBSI was −2.8%. The number of patients with increased ΔBSI was 15 (48.4%) including five patients who received concomitant treatment with ABI or ENZ. In contrast, the number of patients with decreased ΔBSI were 16 (51.6%) including 4 patients who received concomitant treatment with ABI or ENZ.

**FIGURE 4:**
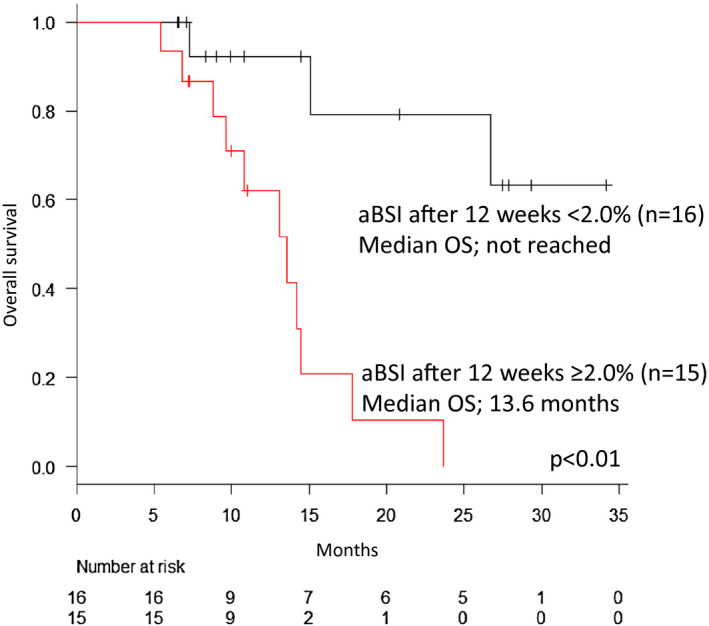
Kaplan‐Meier curve for overall survival (OS) among men treated with Radium‐223 who received a bone scan 12 weeks after treatment with respect to aBSI levels. (n = 31). Median OS in men with < 2.0% aBSI after 12 weeks was not reached (n = 16) and 13.6 months in men with ≥ 1.5% aBSI after 12 weeks (n = 15). There was a significant OS difference between men with < 2.0% aBSI after 12 weeks and men with ≥ 2.0% aBSI after 12 weeks (*P* < .01)

Both univariate and multivariate analysis indicated that aBSI at 12 weeks after first cycle of Ra‐223 administration was a prognostic factor for OS (univariate analysis; ≥2.0% vs < 2.0%, HR 10.20, 95.0%CI 2.19‐47.57, *P* < .01, multivariate analysis; ≥2.0% vs < 2.0%, HR 13.07, 95.0%CI 2.61‐65.53, *P* < .01) (Table 3). While, we found no significant difference of a prognostic value for ΔBSI (Table 3). Figure 4 showed Kaplan‐Meier curve for OS among men treated with Ra‐223 who received a bone scan 12 weeks after treatment with respect to aBSI levels. (n = 31). Median OS in men with < 2.0% aBSI after 12 weeks was not reached (n = 16) and 13.6 months in men with ≥ 2.0% aBSI after 12 weeks (n = 15). There was a significant OS difference between men with < 2.0% aBSI after 12 weeks and men with ≥ 2.0% aBSI after 12 weeks (*P* < .01).

**TABLE 3:** Evaluation of both aBSI at 12 weeks after first cycle of installation and ΔBSI (aBSI change from baseline to 12 weeks after) for predicting OS among men treated with radium‐223 by univariate and multivariate analysis

	Univariate				Multivariate			
	*P*‐value	HR	95.0% CI		*P*‐value	HR	95.0% CI	
			Lower	Upper			Lower	Upper
aBSI after 12 weeks (≥2.0% vs < 2.0%)	<.01	10.20	2.19	47.57	<.01	13.07	2.61	65.53
aBSI change after 12 weeks (increase vs decrease)	.75	0.84	0.29	2.42	.20	0.47	0.15	1.48

Abbreviation: aBSI, automated bone scan index.

Ten out of 15 (66.7%) patients had a decreased aBSI at 24 weeks compared to 12 weeks.

## 4 DISCUSSION

The aBSI has been reported to be a prognostic factor for PC bone metastasis in various clinical settings such as hormone‐naïve and castration‐resistant PC.^6^, ^7^, ^8^, ^9^, ^10^, ^11^, ^12^, ^13^, ^14^, ^15^, ^16^, ^17^, ^18^, ^19^, ^20^, ^22^, ^26^, ^28^, ^29^, ^30^ Recently, Armstrong et al^29^ reported the results of a prospective study that confirmed the use of aBSI as a prognostic imaging biomarker in men treated with tasquinimod (10TASQ10) for mCRPC. In this study, the median OS as measured by aBSI quartile was 34.7, 27.3, 21.7, and 13.3 months and the discriminative ability of aBSI (C index) for OS was 0.63. In a multivariable analysis, a higher aBSI was significantly associated with shorter OS (HR, 1.06; 95% CI, 1.01‐1.11; *P* = .03).

Anand et al^23^ analyzed the prognostic value of aBSI in patients treated with Ra‐223 for mCRPC in a retrospective study. They reported that baseline aBSI was significantly associated with OS (*P* = .01), even though PSA levels were not. Moreover, OS for patients with both aBSI and alkaline phosphatase (ALP) decline was significantly associated with longer survival compared with patients exhibiting only reduced ALP (median, 134 weeks vs median, 77 weeks; *P* = .029). They concluded that baseline aBSI and a change in aBSI upon treatment discontinuation was a significant imaging biomarker for OS in men treated with Ra‐223.

Fosbøl et al^31^ also reported that the aBSI was significantly associated with OS among 88 men treated with Ra‐223 for mCRPC in the multivariate analysis.

Using multivariate analysis, we demonstrated that baseline aBSI is a significant prognostic factor for OS and age, time to CRPC, previous treatment with docetaxel, baseline PSA levels, and GS were not, in men treated with Ra‐223. These findings are compatible with those of Anand et al. and Fosbøl et al. In contrast, our study showed that ΔBSI was not associated with OS, whereas aBSI at 12 weeks after treatment was significantly associated with OS by multivariate analysis. We adopted aBSI change at 12 weeks after treatment as the ΔBSI, whereas Anand et al. adopted the aBSI change after discontinuation of Ra‐223 treatment. Bone flare phenomenon after three cycles of Ra‐223 were reported by Isensee et al.^32^ This phenomenon occurred in 3 out of 19 patients (15.8%) treated with Ra‐223 and they concluded that the treatment should not be changed according to bone scan progression after 3 cycles of Ra‐223. In the current study, 10 out of 15 (66.7%) patients had a decreased aBSI at 24 weeks compared to 12 weeks, which could indicate the bone scan flare phenomenon. The reason for a lack of correlation between ΔBSI and OS in our study may be influenced by bone scan flare phenomenon.

Our previous study showed that median ΔBSI after ABI/ENZ or cabazitaxel was + 0.33% and + 23.5%, respectively, while 14/40 (35.0%) and 7/32 (21.9%) patients exhibited ΔBSI decline, respectively.^18^, ^22^ In this study, the median ΔBSI after Ra‐223 was −2.8% and ΔBSI decline was observed in 16/31 (51.6%) patients. Thus, our study indicated that Ra‐223 may have a significant effect on bone metastases compared with ABI/ENZ or cabazitaxel.

As mentioned above, Armstrong et al already established the use of aBSI as a prognostic imaging biomarker in men treated with tasquinimod for mCRPC,^6^ however, the prognostic value in men treated with Ra‐223 remains unclear. In this study, we reported that aBSI could be an imaging prognostic biomarker for patients treated with Ra‐223. While a predictive marker for the effectiveness of Ra‐223 is also needed. It is important to note that our data do not suggest that aBSI could be predictive biomarker.

There were some limitations to our study. First, this study was retrospective and a small number of patients were evaluated over a short observation time. Second, after treatment failure with Ra‐223, therapy was not uniform and various treatments were performed according to the choice of each physician. To confirm the value of aBSI as an imaging biomarker, a prospective planned study will be needed in a larger cohort of patients with a significant observation time.

## 5 CONCLUSIONS

Baseline aBSI may be a significant prognostic factor for OS in bone metastatic CRPC patients treated with Ra‐223. However, we found no evidence of prognostic value for ΔBSI after 12 weeks of Ra‐223 induction. To confirm of our results, a prospective study in a larger patient cohort with longer observation periods will be needed.

## CONFLICT OF INTEREST

This research received no specific grant from funding agencies in the public, commercial, or not‐for‐profit sectors.

## ETHICS STATEMENT

The experimental procedures were conducted in accordance with the ethical standards of the Helsinki Declaration. This study was approved by the Institutional Review Board of Yokohama City University Medical Center (B181000040).
